# Pharmacokinetic Analysis of Ethanol in a Human Study: New Modification of Mathematic Model

**DOI:** 10.3390/toxics11090793

**Published:** 2023-09-20

**Authors:** Paulo Zekan, Neven Ljubičić, Vladimir Blagaić, Ivan Dolanc, Antonija Jonjić, Miran Čoklo, Alenka Boban Blagaić

**Affiliations:** 1 Department of Obstetrics and Gynecology, Clinical Hospital “Sveti Duh”, Sveti Duh 64, 10000 Zagreb, Croatia; paulo.zekan@gmail.com (P.Z.); vladimir.blagaic@gmail.com (V.B.); 2 Department of Internal Medicine, University Hospital “Sestre Milosrdnice”, Vinogradska cesta 29, 10000 Zagreb, Croatia; neven.ljubicic@kbcsm.hr; 3 Centre for Applied Bioanthropology, Institute for Anthropological Research, Ljudevita Gaja 32, 10000 Zagreb, Croatia; idolanc@inantro.hr (I.D.); antonija.jonjic@inantro.hr (A.J.); mcoklo@inantro.hr (M.Č.); 4 Department of Pharmacology, School of Medicine, University of Zagreb, Šalata 2, 10000 Zagreb, Croatia

**Keywords:** ethanol, expert witnessing, human, mathematic model, pharmacokinetic analysis, Widmark equation

## Abstract

In the pharmacokinetic analysis of ethanol after oral administration, only single- or two-compartment models are used, but their precision in estimating pharmacokinetic parameters might be insufficient. In a recent study, pharmacokinetic analysis using a modified Norberg three-compartment model was performed after oral administration of differently sweetened alcoholic solutions and compared to pharmacokinetic analysis using the classic Widmark model. On three occasions, eight male volunteers consumed differently sweetened alcoholic solutions: non-sweetened, sweetened with sucrose, and sweetened with steviol glycoside. Blood ethanol concentration was determined from samples obtained at t = 15, 30, 60, 90, 120, 180 min after consumption. Pharmacokinetic analysis was performed model independently, using the classic Widmarks model and using the modified Norberg model. Results showed that estimated pharmacokinetic parameters depend on the type of model used. The classic Widmark model in particular overestimated the fraction of absorbed ethanol from the gastrointestinal system to systemic circulation. Furthermore, the type of sweetener also affected pharmacokinetic parameters, although the difference was not statistically significant. In conclusion, the novel pharmacokinetic model, while being more physiological, fits experimental data better and hence is more suitable for modelling real-life alcohol consumption. In addition, the effect of natural non-caloric sweetener steviol glycoside on ethanol pharmacokinetics, analysed for the first time in the current research, might be different when compared to the common-used sweetener sucrose.

## 1. Introduction

### 1.1. Ethanol Pharmacokinetics

Ethanol absorption occurs by the process of passive diffusion through the mucosa of the gastrointestinal system [1]. About 80% of this absorption takes place through the lining of the small intestine and the rest through the lining of the stomach. Therefore, the rate of ethanol absorption is mainly controlled by the passage of gastric content into the intestine, a process regulated by the pyloric sphincter. 

Ethanol is absorbed from the gastrointestinal mucosa into the portal circulation and is therefore subject to first-pass metabolism in the liver before entering the systemic circulation. About 20% of orally consumed ethanol is thought to be metabolized by the first-pass metabolism [2]. Moreover, experimental studies have not yielded unambiguous conclusions about the major site responsible for ethanol first-pass metabolism. Namely, after discovering of the expression of the enzyme alcohol dehydrogenase (ADH) on the gastric mucosa, scientific papers were published indicating that the stomach is also responsible for the first-pass metabolism [3,4]. 

After absorption and first-pass metabolism, the remaining ethanol fraction is distributed in the blood, but also in other tissues such as the brain or skeletal muscle. Since ethanol is a small and hydrophilic molecule, its volume of distribution depends on total body water (TBW). Therefore, in studies, the dose of ethanol administered should be adjusted to either TBW or body weight so that the effects of ethanol on the body can be adequately assessed [5]. The total body water, and thus the volume of ethanol distribution, depends on gender and age. According to the literature, the volume of distribution of ethanol is 0.6 L/kg in women and 0.7 L/kg in men [6]. 

The major elimination pathway of ethanol (90–98%) occurs in the liver through oxidation to acetaldehyde. Other elimination pathways include conjugation of ethanol with the glucuronide and sulphate molecules and excretion of the resulting products in the urine, as well as excretion of non-metabolized ethanol in urine, exhalation, and sweat [5,7]. The oxidative metabolism of ethanol to acetaldehyde involves two enzyme systems. The first enzyme, ADH, is found in the cytoplasm of hepatocytes and metabolizes 85% of ethanol. The other is CYP2E, whose expression is induced by frequent consumption of alcohol, which explains higher ethanol elimination rates in frequent consumers. The range of the blood ethanol elimination rate is 0.10–0.25 g/(L × h) [8,9,10].

### 1.2. Factors Affecting Ethanol Absorption

Gastric emptying is the single most important factor controlling the absorption of ethanol from the gastrointestinal system into the bloodstream [5]. There is no unequivocal opinion in the literature on the mechanism by which gastric emptying affects ethanol absorption. Some researchers emphasize the importance of gastric ADH, which metabolizes ethanol even before its absorption into the portal circulation. On the other hand, by slowing down gastric emptying, the inflow of ethanol into the portal circulation or liver is slowed down. Due to the saturation kinetics of ADH, under the conditions of slow gastric emptying, the fraction of ethanol that is degraded in first-pass metabolism in the liver increases [11]. 

The presence of food in the stomach, that is, the consumption of alcohol together with food, slows down gastric emptying, and affects the concentration of ethanol in the blood. Several studies have shown that, in the presence of food, the maximum concentration of ethanol in the blood, the time to reach the maximum concentration, and the area under the concentration curve decrease [12,13,14].

The non-caloric sodas have increasingly been used in alcohol-based cocktail preparation. Moreover, it is known that the concentration of ethanol in the blood after consuming an alcoholic cocktail with low-caloric juice is higher than after consuming the same amount of ethanol in a cocktail sweetened with sucrose (standard sweetener), and this effect is attributed to gastric emptying [15,16,17]. In all the studies conducted so far on the effects of low-calorie sweeteners on blood ethanol concentration, artificial sweeteners such as cyclamate and aspartame have been used, and we have not found a single study using a natural low-calorie sweetener such as steviol glycoside.

Since the liver is the main organ of ethanol elimination, increased blood flow through the liver leads to faster elimination of ethanol from the body [10]. Consumption of a caloric meal increases blood flow through the portal vein by 52–107%, and thus the consumption of alcohol with a meal can lead to a decrease in the concentration of ethanol in the blood due to increased elimination in the liver [18].

### 1.3. Mathematical Modelling of Ethanol Pharmacokinetics

A detailed explanation of the mathematical modelling of absorption, distribution, and elimination of ethanol is given in Appendix A.

#### Commonly Used Mathematical Models of Ethanol Pharmacokinetics

**Figure 1:**
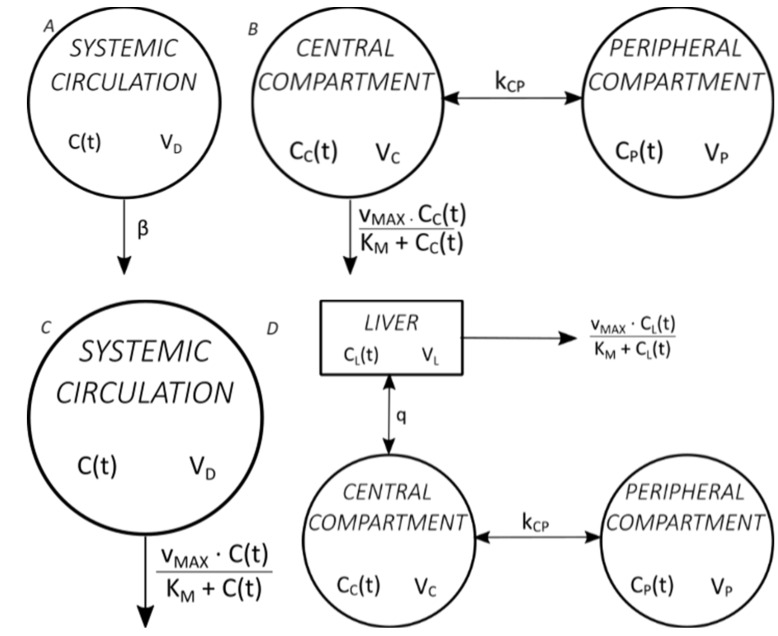
Commonly used mathematical models of ethanol pharmacokinetics. (**A**) The classic Widmark model, with one distribution compartment and elimination by zero-order kinetics [20], (**B**) the Norberg model, with two distribution compartments and elimination by Michaelis–Menten kinetics [21], (**C**) the Wilkinson model, with one distribution compartment and elimination by Michaelis–Menten kinetics [22], (**D**) the Norberg model, with two distribution compartments and liver as a separate compartment and elimination by Michaelis–Menten kinetics from the liver [23].

Figure 1 shows four commonly used mathematical models of ethanol pharmacokinetics [19].

Pharmacokinetic parameters obtained using pharmacokinetic models differ depending on the type of model used. Most ethanol pharmacokinetic studies are used to determine the volume of distribution and elimination parameters (*β*, *K_M_*, *v_MAX_*) so that ethanol is administered intravenously. Although mathematical models of ethanol absorption from the gastrointestinal system have been described and, although ethanol is administered orally in everyday life, only simple single- or two-compartment models are used in the mathematical modelling of oral ethanol administration [10,24]. The Norberg model with three compartments (two compartments of distribution and the liver as a separate compartment) is also called semi-physiological because it describes the ethanol pharmacokinetics most accurately of all models. However, the disadvantage of its use is that it determines pharmacokinetic parameters after intravenous administration [25].

Furthermore, some studies with more physiological models were recently published. Crowell et al. published a physiologically based pharmacokinetic model for ethyl acetate and ethanol but following intravenous application and inhalation exposure [26]. Pastino et al. published a seven-compartment model of ethanol pharmacokinetics in mice [27]. Sultatos et al. developed a computational eight-compartment model but did not validate it in a human study [28]. Umulis et al. conducted a validation of their five-compartment physiological model but using experimental data from previous studies [29].

### 1.4. Forensic Relevance

Driving under the influence of alcohol is a worldwide problem, often with devastating consequences, thus representing an important societal, legal, and forensic challenge. Therefore, such cases often require expert witnessing in court. Expert witnesses are frequently asked to perform a retrograde calculation of blood ethanol concentration at the time in question (accident or traffic stop) based on the result of the chemical analysis or to predict a blood ethanol concentration earlier in time based on a drinking scenario offered by a driver in question.

Most of the expert witnesses in such cases routinely use the Widmark equation, based on elimination rates and a factor “r”, also known as the “Widmark factor”. Widmark calculated this factor based on a study from 1932 performed on several men and women, by calculating average values for the elimination rate and r. Over time, improvements to the Widmark equation have been made and new scientific findings on the pharmacology of alcohol have been published, including alternative models, such as the one we propose in this paper. However, in everyday forensic practice, these improvements are often ignored without any rational and valid explanation. This becomes problematic in many instances, especially if we consider the fact that drivers under the influence of alcohol are often subject to serious financial fines, losing driving privileges, and sometimes even arrest and imprisonment, with all the legal, economic, and social consequences. Therefore, alternative models definitely should have their place in forensic practice and expert witnessing.

### 1.5. Aims of the Study

In the literature, we did not find any study in which the Norberg three-compartment model was used after oral administration of ethanol. Adaptation of the Norberg model for oral administration would allow a better estimate of the absorbed ethanol fraction. Therefore, the first aim of the study was to conduct a pharmacokinetic analysis from our experimental data using a novel, physiologically based Norberg model adapted for oral administration and to compare the results with the pharmacokinetic analysis using the classic Widmark model. 

Having become enormously popular, natural non-caloric sweeteners, like steviol glycoside, have also been used in beverage production. Although its effect on ethanol pharmacokinetics can be deduced from previous publications with other non-caloric sweeteners, we could not find any human research studying the effects of natural non-caloric sweetener steviol glycosides on ethanol pharmacokinetics. Hence, the second aim of the study was to determine the effect of sweeteners (sucrose and steviol glycoside) on the pharmacokinetic parameters of ethanol using both classic and novel physiologically based models and to compare them depending on the type of sweetener.

## 2. Materials and Methods

### 2.1. Study Design

The study involved 8 healthy male volunteers (age: 24–25, body weight: 82–112 kg). This is heavier than the “reference man” (cca 73 kg), but due to the fact that we took their body weight into consideration (we used ethanol by g/kg of body weight) it does not bias the study and the developed model and parameters. All volunteers completed a questionnaire about their health before the start of the study. All subjects consumed no more than one alcoholic beverage weekly, did not consume any alcoholic beverages 48 h before the study, did not have a family or personal history of alcohol-induced metabolic disorder, and did not have a family or personal history of addiction to substances including alcohol. Each of the 8 subjects consumed alcoholic beverages sweetened with various sweeteners on three occasions at intervals of at least three days and three hours after their last meal. The alcoholic beverages consumed were ethanol solutions sweetened with various sweeteners: unsweetened control solution (C), solution sweetened with high-caloric sweetener sucrose (SU), and solution sweetened with the low-caloric natural sweetener steviol glycoside (ST). The study was blinded on the type of beverage consumed and the order of consumption was randomly determined. 

### 2.2. Ethanol Solution Preparation

Subjects consumed 0.3 L of a solution containing 0.3 g/kg (0.3 g per kg body weight) of pure ethanol. The solutions were prepared so that 0.93 mL/kg of Johnnie Walker Red Label^®^ (*v*/*v* = 40%) was dissolved in tea (sweetened with various sweeteners) at room temperature to a total volume of 0.3 L. The ethanol concentration in whiskey was validated prior to the experiment. Group C tea was unsweetened. The SU group tea was prepared so that 112.5 g of table sugar (Šećer Kristal^®^, Konzum d.d., Zagreb, Croatia) was dissolved in 1 L of tea. The ST group tea was prepared according to the instructions of the manufacturer so that its sweetness corresponds to SU group tea, i.e., 38 tablets of steviol glycoside (Sussina Stevia^®^, INSTANTINA Ges.m.b.H., Dürnkrut, Austria) were dissolved in 1 L of tea.

### 2.3. Ethanol Solution Consumption and Blood Sampling

Before the start of the study, all subjects had an intravenous catheter through which blood samples were taken. The blood ethanol concentration obtained before ethanol solution consumption was the blood ethanol concentration at t = 0. Subjects had to drink the prepared solution in 2 min. Samples of venous blood were taken at time points t = 15, 30, 60, 90, 120, and 180 min after the end of consumption. All subjects were provided with a high-calorie meal upon completion of the study.

### 2.4. Blood Analysis

Blood was collected in plain tubes without additives (VACUETTE^®^ Z Serum Sep Clot Activator^®^, Greiner Bio-One Italia S.r.l., Cassina de’ Pecchi, Italy). Samples were left to clot for 1 h at room temperature and then centrifuged for 10 min at 10,000 rpm on an Eppendorf^®^ MiniSpin^®^ centrifuge. Tubes of blood were kept closed at all times and in a vertical position. Sera were immediately separated from the cel–s-using transfer pipets, the supernatant from each tube was transferred to a sample tube. Samples were analysed without delay and immediately after opening the sample tube. Precaution measures were taken to prevent alcohol evaporation from calibrators, controls, and samples. The blood ethanol concentration was determined from serum using the automated enzymatic assay of ADH with spectrophotometric analysis of the final product (Beckman Coulter AU 680^®^, Beckman Coulter Inc., Brea, CA, USA). The principle of reaction is that ADH catalyses the oxidation of ethanol to acetaldehyde with the concurrent reduction of NAD+ to NADH. The system monitors the rate of change in absorbance due to NADH at 340 nm. The rate of change in absorbance due to NADH is directly proportional to the concentration of ethanol in the sample and is used by the system to calculate and express the ethyl alcohol concentration based upon a two-point calibration curve [30]. The reagents for the analysis were produced by Microgenics Corporation, Fremont, CA, USA. The analysis was performed at the Department for Medical Laboratory Diagnostics, University Hospital “Sveti Duh”, Zagreb, Croatia.

### 2.5. Pharmacokinetic Analysis

Blood ethanol vs. time curves were obtained for each subject and were the basis for further pharmacokinetic analysis. The analysis was performed in three ways: model independently, using the classic Widmark model, and using the modified Norberg three-compartment model.

#### 2.5.1. Model-Independent Pharmacokinetic Analysis

The pharmacokinetic parameters determined were blood ethanol concentration at 15 min (C_15_), maximum blood ethanol concentration at a period from 0 to 180 min (C_MAX_), and area under the blood ethanol concentration vs. time (AUC). AUC was determined using the trapezoid rule.

#### 2.5.2. Pharmacokinetic Analysis Using the Classic Widmark Model

Working with the classic Widmark model we estimated the blood ethanol elimination rate (*β*) as well as the fraction of ethanol absorbed from the gastrointestinal system to systemic circulation (*f*_1_). The model is based on the following assumptions:

1. The dose of administered ethanol (*D*) immediately reaches the site of absorption (gastrointestinal system) from where only its fraction, namely *f*_1_, is instantly absorbed into the systemic circulation.
2. Ethanol is distributed in the body in a single compartment corresponding to ethanol´s volume of distribution (*V_D_*).
3. Ethanol is eliminated following zero-order kinetics with rate *β*.

**Figure 2:**
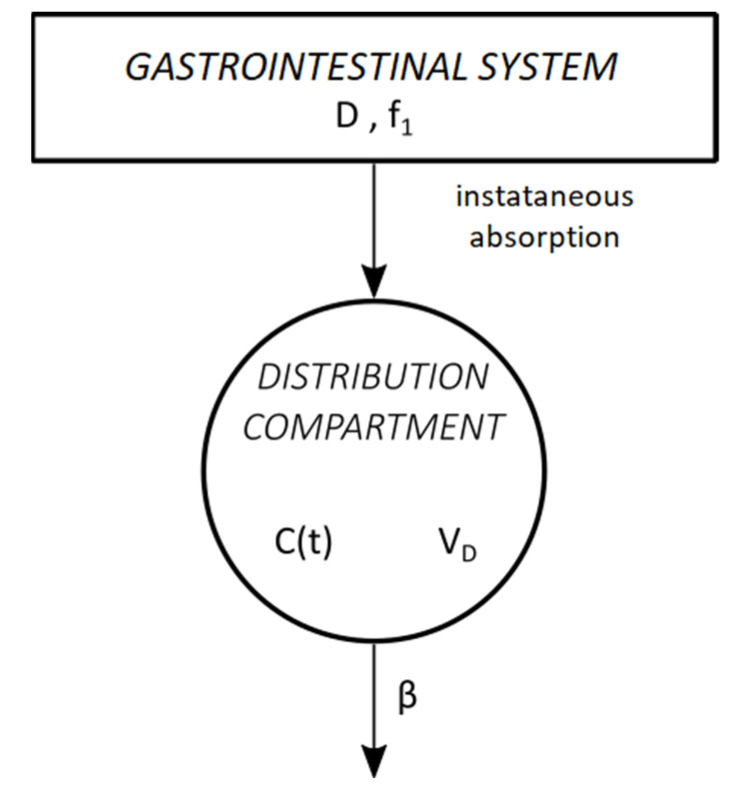
Schematic representation of the classic Widmark model. Ethanol is instantly absorbed from the gastrointestinal system into the distribution compartment from where it is eliminated by zero-order kinetics. *D*: dose of administered ethanol (g); *f*_1_: fraction of absorbed dose; *C*(*t*): concentration of ethanol in the distribution compartment at time t (g/L); *V_D_*: volume of distribution (L); *β*: elimination constant of ethanol from the distribution compartment (g/(L × min)).

A schematic representation of the classic Widmark model is given in Figure 2.

The amount of absorbed ethanol is given by:

$$f_{1}\cdot D$$

Hence, *C*_0_ (extrapolated ethanol concentration at time *t* = 0) is given by

$$C_{0}=\frac{f_{1}\cdot D}{V_{D}}$$

Time-dependent change of blood ethanol concentration is given by the following differential equation:

$$\frac{dC}{dt}=-\beta ,\;C\left(0\right)=C_{0}$$

The solution of the aforementioned differential equation is underneath the linear equation.

$${C\left(t\right)=C}_{0}-\beta t$$

Values of *C*_0_ and *β* were determined from the experimental data as follows: Least square linear regression line was obtained from blood ethanol concentrations at *t* = 60, 90, and 120 min (RStudio, Version 1.1.383-© 2009–2017 RStudio, Inc., Boston, MA, USA); *C*_0_ was determined as the y-intercept of the line (blood ethanol concentration), and *β* as the negative value of the slope of the line. The literature value of the distribution volume (*V_D_* = 0.7 L/kg) was used in the model. Therefore, we determined the fraction of absorbed ethanol from the following equation:

$$f_{1}=\frac{C_{0}\cdot V_{D}}{D}$$

#### 2.5.3. Pharmacokinetic Analysis Using the Modified Norberg Three-Compartment Model

##### Structural Model

Norberg’s three-compartment model modified for oral administration was used to estimate the fraction of absorbed ethanol from the gastrointestinal system into the systemic circulation (*f*_1_) and to estimate the value of the first-order absorption constant (*k*_*A*_). The following assumptions were used in this model:

1. The dose of administered ethanol (*D*) immediately reaches the site of absorption (gastrointestinal system), from where only its fraction *f*_2_ is absorbed into the portal circulation (liver) by a first-order process with absorption constant *k*_*A*_.
2. The gastrointestinal system is modelled as a single compartment.
3. Ethanol is eliminated in the liver following Michaelis–Menten kinetics, and the liver is modelled as a separate compartment.
4. Ethanol is distributed in two compartments (central and peripheral).

**Figure 3:**
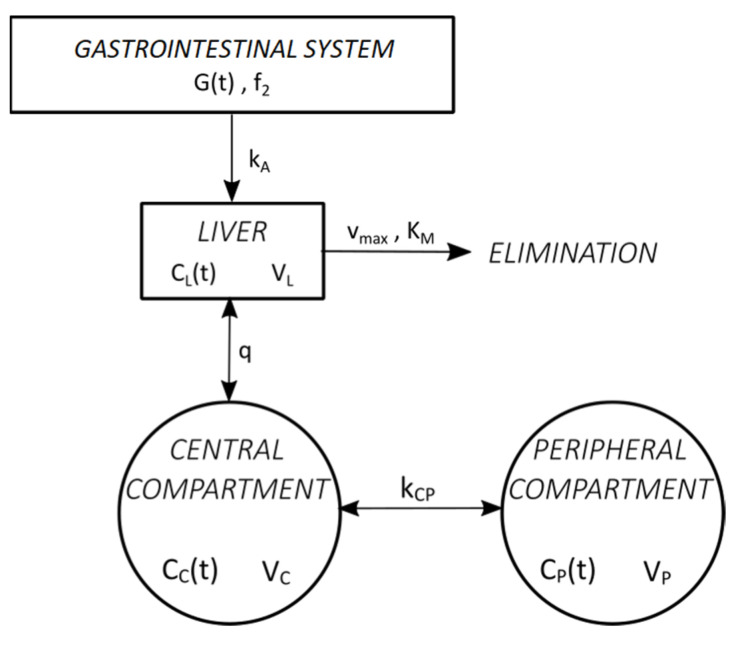
Schematic of the novel model. Here, *f*_2_: fraction of absorbed ethanol; *G(t):* amount of ethanol remaining in the gastrointestinal system at time *t*; *k_A_*: first-order absorption constant (min^−1^); *C_C_(t):* central compartment ethanol concentration (g/L); *C_P_(t)*: peripheral compartment ethanol concentration (g/L); *C_L_(t)*: liver compartment ethanol concentration (g/L); *V_C_*: central compartment volume (L); *V_P_*: peripheral compartment volume (L), *V_L_*: liver compartment volume (L); *k_CP_*: ethanol central–peripheral distribution constant (L/min); *q*: liver blood flow (L/min); *v_MAX_*: ethanol maximum elimination rate (g/min); *K_M_*: Michaelis–Menten constant (g/L).

A schematic representation of the Norberg three-compartment model adapted for oral administration is shown in Figure 3.

Absorption of ethanol from the gastrointestinal to the liver compartment is a first-order process given by the following equation:

$$\frac{dG}{dt}=-k_{A}\cdot \left(G\left(t\right)-\left(1-f_{2}\right)\cdot G\left(0\right)\right)\;,\;G\left(0\right)=D$$

Time-dependent change in *C_L_* due to absorption of ethanol from the gastrointestinal system is:

$$-\frac{1}{V_{L}}\cdot \frac{dG}{dt}$$

Time-dependent change in *C_L_* due to ethanol elimination in the liver is:

$$-\frac{v_{MAX}\cdot C_{L}\left(t\right)}{{V_{L}\cdot (K}_{M}+C_{L}\left(t\right))}$$

Time-dependent change in *C_L_* due to blood flow between liver and central compartment is:

$$\frac{q\cdot (C_{C}\left(t\right)-C_{L}\left(t\right))}{V_{L}}$$

Hence, *C_L_* is given by the following first-order differential equation:

$$\frac{dC_{L}}{dt}=\frac{k_{A}\cdot \left(G\left(t\right)-\left(1-f_{2}\right)\cdot G\left(0\right)\right)}{V_{L}}-\frac{v_{MAX}\cdot C_{L}\left(t\right)}{{V_{L}\cdot (K}_{M}+C_{L}\left(t\right))}+\frac{q\cdot (C_{C}\left(t\right)-C_{L}\left(t\right))}{V_{L}}$$

Time-dependent change in *C_C_* due to blood flow between the central compartment and peripheral compartment is:

$$\frac{q{\cdot (C}_{L}\left(t\right)-C_{C}\left(t\right))+k_{CP}{\cdot (C}_{P}\left(t\right)-C_{C}\left(t\right))}{V_{C}}$$

Hence, *C_C_* is given by the following first-order differential equation:

$$\frac{dC_{C}}{dt}=\frac{q{\cdot (C}_{L}\left(t\right)-C_{C}\left(t\right))+k_{CP}{\cdot (C}_{P}\left(t\right)-C_{C}\left(t\right))}{V_{C}}$$

Similarly, *C_P_* is given by the following first-order differential equation:

$$\frac{dC_{P}}{dt}=\frac{k_{CP}{\cdot (C}_{C}\left(t\right)-C_{P}\left(t\right))}{V_{P}}$$

Thus, the modified Norberg model is represented with a system of four first-order differential equations:

$$\frac{dG}{dt}=-k_{A}\cdot \left(G\left(t\right)-\left(1-f_{2}\right)\cdot G\left(0\right)\right)\;$$

$$\frac{dC_{L}}{dt}=\frac{k_{A}\cdot \left(G\left(t\right)-\left(1-f_{2}\right)\cdot G\left(0\right)\right)}{V_{L}}-\frac{v_{MAX}\cdot C_{L}\left(t\right)}{{V_{L}\cdot (K}_{M}+C_{L}\left(t\right))}+\frac{q\cdot \left(C_{C}\left(t\right)-C_{L}\left(t\right)\right)}{V_{L}}$$

$$\frac{dC_{C}}{dt}=\frac{q{\cdot (C}_{L}\left(t\right)-C_{C}\left(t\right))+k_{CP}{\cdot (C}_{P}\left(t\right)-C_{C}\left(t\right))}{V_{C}}$$

$$\frac{dC_{P}}{dt}=\frac{k_{CP}{\cdot (C}_{C}\left(t\right)-C_{P}\left(t\right))}{V_{P}}$$

Initial conditions of the system are:

$$G\left(0\right)=D\;,\;\;C_{L}\left(0\right)=0,\;\;C_{S}\left(0\right)=0,\;\;C_{P}\left(0\right)=0$$

##### Statistical Model

All experimental data were simultaneously fitted using non-linear mixed effect modelling with the Monolix-2023R1 (Lixoft©, Antony, France) software using the structural model given above. Parameter estimation was performed using the stochastic approximation expectation maximization (SAEM). Individual model parameters were acquired *post hoc* using the mean of the full posterior distribution.

In the Monolix-2023R1 (Lixoft©) software, statistical models consist of observational and individual models. The observational model was a proportional error model with a normal distribution of the residual error parameter and was given as follows:

$$C_{i,t}=c_{i,t}+a\times c_{i,t}\times \varepsilon_{i,t}$$

*C_i_*_,*t*_ is observed blood ethanol concentration for individual *i* at time *t*, *a* is proportional error model constant, and *ε_i_*_,*t*_ is random variable from a normal distribution with mean 0.

Individual models were separately given for each parameter:

$$\log{k_{a}}_{i}=\log{k_{a}}_{pop}+\eta_{{k_{a}}_{i}}$$

$$\log{f_{2}}_{i}=\log{f_{2}}_{pop}+\eta_{{f_{2}}_{i}}$$

$${v_{MAX}}_{i}={v_{MAX}}_{pop}$$

$${k_{CP}}_{i}={k_{CP}}_{pop}$$

where *k_a i_*, *I_2 i_*, *v_MAX i_*, and *k_CP i_* are estimated values of parameters for individual *i* where *k_a pop_*, *p_pop_*, *v_MAX pop_*, and *k_CP pop_* are estimated population values. *η_ka i_* and *η_f 2 i_* are random variables from a normal distribution with mean 0. Hence, all inter-individual variability for pharmacokinetic parameters were modelled using a log-normal distribution.

We did not include random effects for *v_MAX_* and *k_CP_* in our model. In order for the statistical model not to be over parametrized, values of other pharmacokinetic parameters were taken as constants and included in the model as regressor variables (Table 1).

The model was evaluated with an observation vs. predictions plot, the precision of the parameter estimate was given as the relative standard error and the Akaike Information Criterion (AIC). The lower AIC values correspond to a better model fit.

Group, i.e., type of sweetener used, was included in the statistical model as categorical covariate. We used an ANOVA test (as implemented in Monolix) to test whether covariates should be added to the model. Furthermore, visual inspection of random effect scatterplots as well as values of the Pearson coefficient of correlation were used to look for correlations between model parameters. Finally, inclusion of both covariates and correlation estimates in the final model depended on the new value of AIC and the precision of the parameter estimates.

### 2.6. Statistical Analysis

Microsoft Excel^®^ was used for data collection. Values of all pharmacokinetic parameters were compared between the groups using the Kruskal–Wallis test with the post hoc Wilcoxon test. Values of *f*_1_ and *f*_2_ within the same group were compared using the Wilcoxon test (RStudio, Version 1.1.383–© 2009–2017 RStudio, Inc.). Statistical modelling of the novel model was performed in Monolix–2023R1 (Lixoft©). Data were given as a mean with a 95% confidence interval (CI) for pharmacokinetic parameters obtained using the non-compartmental and the Widmark model analysis and as a point estimate with standard error for pharmacokinetic parameters obtained using non-linear mixed effect modelling for the novel model. A *p*-value of less than 0.05 was considered statistically significant. Concentration vs. time plots were made in RStudio, Version 1.1.383–© 2009–2017 RStudio, Inc.

### 2.7. Informed Consent and Ethical Committee

All subjects signed an informed consent in which the research protocol was explained to them in detail. The research protocol was approved by the Ethical Committee of “BC “Sestre Milosrdnice” (EP-15659/18-9) where the experimental part of the research was conducted.

## 3. Results

A demographic table is shown in Table 2.

**Figure 4:**
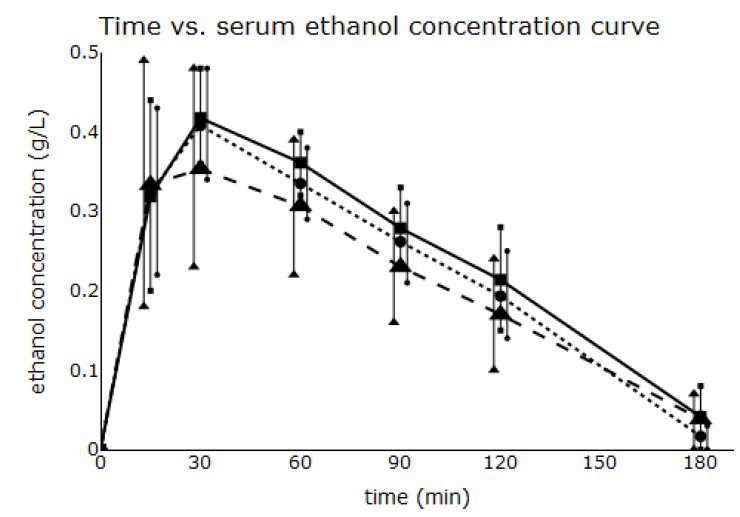
Serum ethanol concentration vs. time curves for all study groups. Data are mean values of the serum ethanol concentration ± standard deviation. ---■--- control ethanol solution (C) - -▲- - sucrose-sweetened ethanol solution (SU) ⋅⋅⋅●⋅⋅⋅ steviol glycoside-sweetened ethanol solution (ST).

All blood samples were successfully analysed for ethanol concentration. Serum ethanol concentration vs. time curves for study groups s are shown in Figure 4.

Values of obtained pharmacokinetic parameters are shown in Table 3 and Table 4 for the model-independent and Widmark model analyses, respectively. 

All pharmacokinetic parameters did not significantly differ between groups. However, values of C_MAX_, AUC, and *f*_1_ were lower in the SU than in the C and ST groups. 

Results of non-linear mixed effect modelling for the novel model analysis are shown in Table 5. 

Correlation between random effects was low (*r* = −0.023), hence the correlation between random effects (*k_A_* and *f*_2_) was not included in the final model. Furthermore, covariate “group” (i.e., type of sweetener used) did not have significant effect on *k_A_* and *f*_2_. Akaike Information Criteria (AIC) was calculated to be −1607.86, providing satisfactory model fitting. We also calculated the AIC value for Widmark’s model using the same methodology in Monolix and it was calculated to be −215.55. 

Results of pharmacokinetic parameter individual estimates compared by group are shown in Table 6. Pharmacokinetic parameters expectedly did not significantly differ between groups.

**Figure 5:**
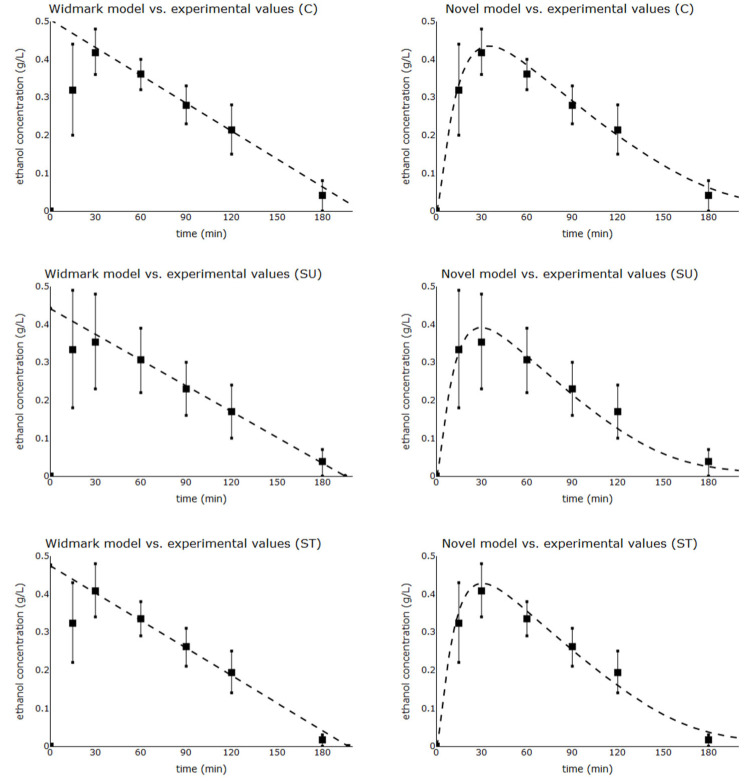
Modelled values of serum ethanol concentration (Widmark and novel models) and experimental data (mean ± standard deviation) are shown separately for each study group (N = 8). ■ Experimental data (serum ethanol concentration mean)-modelled values, C = control, SU = sucrose, ST = steviol glycoside.

Comparison between experimental data and modelled values of serum ethanol concentration is shown in Figure 5.

**Figure 6:**
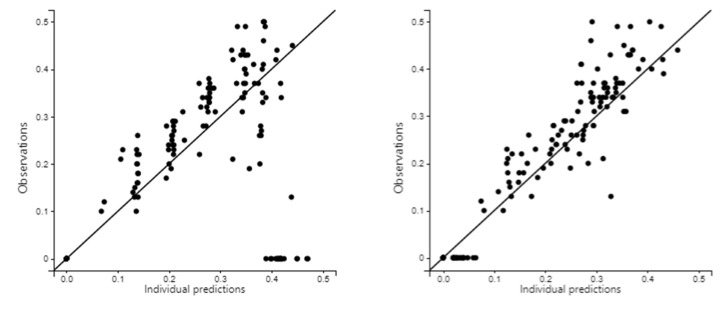
Individual prediction vs. observation scatterplots. Left picture—Widmark’s model. Right picture—novel model.

Individual prediction vs. observation scatterplots measure goodness of fit for each model and are shown in Figure 6. They show that the novel model predicts the ethanol pharmacokinetics better than Widmark’s model. However, the novel model is imprecise, especially at higher ethanol concentrations.

## 4. Discussion

The comparison of the novel model with recently published models is given in Table 7.

Established values of the ethanol elimination rate *β* from Widmark´s model (0.0023–0.0025 g/(L × min)) are in accordance with literature values (0.0014–0.0028 g/(L × min)) obtained in research using comparable doses of ethanol [31,32]. 

We used a nonlinear mixed effect modelling approach to develop our novel model, which provided us with a better understanding of the source of variability. Moreover, this approach allowed us to better diagnose the model fits and compare them in that sense.

Furthermore, *k_A_* has greater values in our research (0.08 min^−1^) when compared to other studies of ethanol absorption (0.01–0.06 min^−1^), implying ethanol absorption to portal circulation was faster in our research [33]. We believe the application of the novel model might explain the difference since values of pharmacokinetic parameters are dependent on the type of model used. Moreover, we gave our subjects lower ethanol doses than those in other research, so our results support previous findings of faster ethanol absorption at lower doses [10]. Finally, high value of relative standard error for k_a_ implies imprecision of our estimate. 

Unlike other mathematical models which estimate the fraction of ethanol eliminated through first-pass metabolism (gastric and liver first-pass metabolism jointly), our model enables the estimation of the fraction which is absorbed in the portal circulation (gastric first-pass metabolism separately). Calculated values of *f*_2_ (0.49–0.52) imply ethanol absorption was incomplete. In our opinion, the incomplete absorption can be explained by the low ethanol dose and short lag time after the last meal. Most ethanol pharmacokinetic studies used doses greater than 0.5 g/kg and administered it more than 10 h after the last meal [10], unlike to the current study where the ethanol dose was 0.3 g/kg and was administered 3 h after the last meal. Hence, the results agree with previous experiments about the role of gastric ADH in the ethanol first-pass metabolism, which was found to be greater with lower ethanol doses due to ADH saturation kinetics [3,4]. Furthermore, our findings favourably correlate with studies about the effect of food on ethanol pharmacokinetics, which showed simultaneous consumption of food and ethanol lowered blood ethanol concentration [12,13,14,31].

However, the estimated fraction of absorbed ethanol depends on the values of V_C_ and V_P_ used in the model. Since we did not experimentally obtain those values, this estimation might be imprecise. However, we still believe the novel pharmacokinetic model, being more physiological, might simulate real-life alcohol intake more precisely. 

The difference in absorbed fractions of ethanol between models, *f*_1_ > *f*_2_, was statistically significant and might imply an overestimation of the absorbed fraction of ethanol using the Widmark model. The observed difference is due to the fact that the Widmark model estimates ethanol concentration at t = 0 as the y-intercept after retrograde extrapolation of linearly modelled data, which is obviously an overestimation of the real situation. Furthermore, the area under the Widmark curve is greater than the area under the curve in the novel model. Consequently, the estimated absorbed fractions are different since the area under the curve is a common pharmacokinetic parameter in estimating the absorbed fraction of a substance.

This finding might be considered in forensic analysis of ethanol when the Widmark model is used, especially in the estimation of blood ethanol concentration in cases of drunken driving. Widmark’s equation might not be applicable in cases of consuming a low dose of alcohol shortly before the accident. In addition, intra-individual and inter-individual variations must be taken into consideration when expert witnessing such cases and using retrograde extrapolation to estimate blood ethanol concentration at an earlier point in time. It is obvious that the “one suit for all occasions” approach is not optimal in such cases and that they require a certain level of a “personalised” approach. Therefore, alternative models, such as the one we are proposing, should be considered in specific situations (as previously described), no matter if they might be mathematically more challenging than the “simplified” one-for-all approach.

Our research showed that pharmacokinetic parameters were not affected by the type of sweetener, irrespective of the model used for analysis. Although C_MAX_, AUC, *f*_1_, and *f*_2_ were lower in the sucrose group, the difference was not statistically significant in comparison to other groups. Hence, those results were not supported by previous research which found a change in pharmacokinetic parameters after the consumption of alcoholic beverages sweetened with low-caloric artificial sweeteners (cyclamate or aspartame) in comparison to alcoholic beverages sweetened with sucrose. [15,16,17]. We believe our findings can be explained by the low dose of ethanol used and the relatively short time lag between the last meal and ethanol consumption since ethanol absorption is known to be prone to intraindividual variability under such circumstances [12,19]. 

Our research might have a few limitations. We only estimated the absorbed fraction of ethanol but did not measure it directly. Therefore, the true validation of our model was impossible. Furthermore, we did not experimentally obtain TBW and percentage of fat for each individual in order to estimate *V_D_*, *V_C_*, and *V_P_* more appropriately. Since all calculated values depend on those parameters, further research is needed to establish proper values of the parameters when the novel model is used. Our study is a pilot project and requires further research by expanding it to other age groups, women, and a larger research group. This would enable us to estimate a larger number of pharmacokinetic parameters and each of them more precisely.

The strength and novelty of our research lies in the fact that we presented novel human pharmacokinetic data regarding sweetener treatments and comparison of their influence on the pharmacokinetics of ethanol, particularly since the conventional hypothesis is that artificial sweeteners increase absorption rates. 

Comparing the AIC values calculated for both the novel and Widmark models, we can conclude that our novel model showed much better model fitting (−1607.86 vs. −215.55). 

## 5. Conclusions

This research studied ethanol pharmacokinetics after oral administration of ethanol using a novel mathematical model. The results suggest superiority of the novel model to the classic Widmark model. In addition, absorption is shown to be a critical step in ethanol pharmacokinetics. Furthermore, steviol glycoside, whose influence was also investigated for the first time, does not affect ethanol pharmacokinetics when used with low doses of ethanol and shortly after the last meal. 

The significance of this research is an obvious forensic analysis of ethanol, especially in the estimation of blood ethanol concentration in specific cases of drunken driving, for example, when a hip flask defence (stating that a driver had consumed alcohol between the time of an accident and a breathalyser test, so that a positive result does not actually indicate drunken driving) is being used. Alternative models, such as the one we are proposing, should be considered, especially in cases of consuming low doses of alcohol shortly before the accident, no matter if they might be mathematically more challenging than the “simplified” one-for-all approach, such as the Widmark model.

## Figures and Tables

**Table 1:** Values of constants used in the novel model.

Constant	Value	Unit
ethanol dose/kg	0.3	g
*V_L_*	2.2	L
*Q*	1.1	L/min
*K_M_*	0.02	g/L
*V_D_*/kg	0.7	L
*V_C_*/kg	0.28	L
*V_P_*/kg	0.42	L

**Table 2:** Demographic table.

	Median	Interquartile Range
N	8	
Age (years)	24	1
Body weight (kg)	93	5.25
Height (cm)	185	2.75
**Sex**		
Male	8	
Female	0	

**Table 3:** Values of pharmacokinetic parameters for all study groups obtained by model-independent analysis. N: number of subjects in the group, C_15_: serum ethanol concentration 15 min after consumption, C_MAX_: maximum serum ethanol concentration over period of 180 min after consumption, AUC: area under serum ethanol concentration vs. time curve over a period of 180 min after consumption. The *p*-values are obtained by the Kruskal–Wallis test (C vs. SU vs. ST). * statistically significant *p*-value. Data are given as mean with 95% CI in parentheses.

Pharmacokinetic Parameters	Control (C) N = 8	Sucrose (SU) N = 8	Steviol Glycoside (ST) N = 8	*p*-Value (C vs. SU vs. ST)
C_15_ (g/L)	0.32 (0.22–0.42)	0.33 (0.21–0.46)	0.32 (0.30–0.38)	0.9153
C_MAX_ (g/L)	0.42 (0.38–0.47)	0.37 (0.25–0.49)	0.41 (0.33–0.49)	0.8597
AUC ((g × min)/L)	0.32 (0.22–0.42)	0.33 (0.21–0.46)	0.32 (0.30–0.38)	0.9153

**Table 4:** Values of pharmacokinetic parameters for all study groups obtained using Widmark’s model. N: number of subjects in the group, *β*: ethanol elimination rate, *f*_1_: fraction of absorbed ethanol calculated using Widmark´s model. The *p*-values are obtained by the Kruskal–Wallis test (C vs. SU vs. ST). Data are given as mean with 95% CI in parentheses.

Pharmacokinetic Parameters		Sucrose (SU) N = 8	Steviol Glycoside (ST) N = 8	*p*-Value (C vs. SU vs. ST)
*β* (g/(L × min))	0.0025 (0.0020–0.0029)	0.0023 (0.0013–0.0032)	0.0024 (0.0019–0.0029)	0.8267
*f*_1_	1.05 (0.97–1.12)	0.93 (0.69–1.16)	1.00 (0.87–1.12)	0.5845

**Table 5:** Population parameter estimates obtained using the non-linear mixed effect modelling with the novel model. Here, *k_A_*: first-order ethanol absorption constant calculated using the novel model, *f*_2_: fraction of absorbed ethanol calculated using the novel model, *v_MAX_*: maximum ethanol elimination rate calculated using the novel model, *k_CP_*: intercompartment distribution constant calculated using the novel model, *a*: proportional error model parameter.

	Point Estimate	Standard Error	Relative Standard Error (%)
	Fixed effects		
*k_A_* (min^−1^)	0.081	0.0077	9.47
*f*_2_	0.51	0.016	3.08
*v_MAX_* (g/min)	0.087	0.00014	0.160
*k_CP_* (L/min)	0.01	0.000078	0.773
	Standard deviations of random effects		
*k_A_*	0.091	0.12	129
*f*_2_	0.099	0.024	24.5
	Proportional error model parameter		
*a*	0.46	0.035	7.72

**Table 6:** Values of pharmacokinetic parameters for all study groups obtained using the novel model. N: number of subjects in the group, *k_A_*: first-order ethanol absorption constant calculated using the novel model, *f*_2_: fraction of absorbed ethanol calculated using the novel model, *v_MAX_*: maximum ethanol elimination rate calculated using the novel model, *k_CP_*: intercompartment distribution constant calculated using the Novel model. The *p*-values are obtained by the Kruskal–Wallis test (C vs. SU vs. ST). * statistically significant *p*-value. Data are given as mean with 95% CI in parentheses.

Pharmacokinetic Parameters	Control (C) N = 8	Sucrose (SU) N = 8	Steviol Glycoside (ST) N = 8	*p*-Value (C vs. SU vs. ST)
*k_A_* (min^−1^)	0.081 (0.079–0.082)	0.082 (0.080–0.083)	0.081 (0.080–0.082)	0.731
*f*_2_	0.520 (0.489–0.551)	0.507 (0.450–0.563)	0.492 (0.459–0.524)	0.360
*v_MAX_* (g/min)	0.087	0.087	0.087	N/A
*k_CP_* (L/min)	0.01	0.01	0.01	N/A

**Table 7:** The comparison of published models with the proposed model. MM: Michaelis–Menten kinetics N/A: Not available * Model estimate values of *β*. ** Value of *v_MAX_* was taken from the literature and was not experimentally calculated. *** This is a computational model which was not tested with experimental data.

Reference	Subject	Route of Administration	Elimination Order	Type of the Model	Number of Compartments	*v_MAX_*	*k_A_*
Widmark et al. [19]	Dogs	Peroral	0	Phenomenological	1	N/A *	N/A *
Wilkinson et al. [22]	Humans	Intravenous	MM	Phenomenological	1	0.16	N/A
Norberg et al. [28]	Humans	Intravenous	MM	Phenomenological	2	0.095	N/A
Norberg et al. [31]	Humans	Intravenous	MM	Semi-physiological	3	0.089	N/A
Umulis et al. [29]	Humans	Peroral	MM	Physiological	5	0.15	0.05
Pastino et al. [25]	Mouse	Intraperitoneal	MM	Physiological	7	N/A **	N/A
Sultatos et al. [23]	Humans	Peroral	MM	Physiological	8	N/A ***	N/A ***
NOVEL MODEL	Humans	Peroral	MM	Physiological	4	0.087	0.08

**Figure A1:**
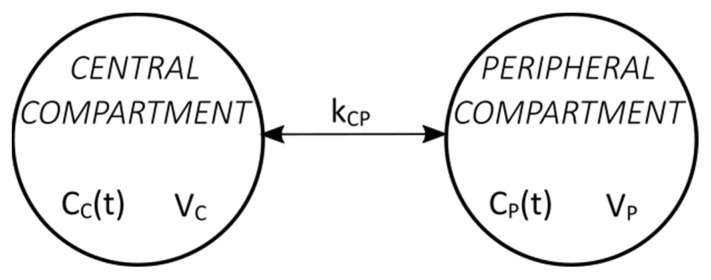
Two-compartment distribution model. *C_C_(t)*: ethanol concentration in the central compartment (g/L), *C_P_(t)*: ethanol concentration in the peripheral compartment (g/L), *V_C_*: volume of the central compartment (L), *V_P_*: volume of the peripheral compartment (L), *k_CP_*: intercompartment distribution constant (L/min).

**Figure A2:**
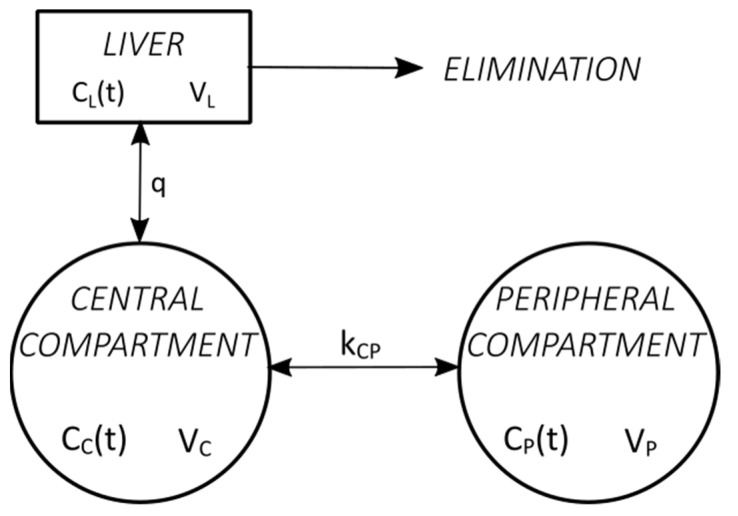
Two-compartment distribution model with liver as a separate compartment. *C_C_(t)*: ethanol concentration in the central compartment (g/L), *C_P_(t)*: ethanol concentration in the peripheral compartment (g/L), *C_L_(t)*: liver ethanol concentration (g/L), *V_C_*: volume of the central compartment (L), V_C_: volume of the central compartment (L), *V_P_*: volume of the peripheral compartment (L), *k_CP_*: intercompartment distribution constant (L/min) *q*: blood liver flow (L/min).

## Data Availability

The data presented in this study are available on request from the corresponding author. The data are not publicly available due to privacy of participants.

## Appendix A

### Appendix A.1. Absorption

The absorption of ethanol through the mucosa of the small intestine into the portal circulation takes place by simple diffusion, so ethanol absorption is usually modelled by a first-order differential equation [2]. However, the absorption of ethanol does not occur immediately after consumption due to the time lag between consumption and absorption. Hence, some researchers have chosen to use an extra first-order model to overcome the problem with the time lag [10]. However, the absorption constant through the small intestine is much higher (half-life is shorter) than the gastric emptying constant, so in pharmacokinetic models, ethanol absorption is often modelled by only one compartment in the digestive system where the common absorption constant combines the gastric emptying and absorption constants. Due to the incomplete absorption of ethanol from the digestive system to the portal circulation, the ethanol absorption model should be adapted for the fraction of ethanol that reaches the small intestine [2]. These models are shown in Table A1.

**Table A1:** Mathematical models of ethanol absorption. *G(t)*: gastric amount of ethanol (g), *I(t)*: intestinal amount of ethanol (g), *k_A_*: first-order absorption constant (min^−1^), *k_G_*: first-order constant of gastric emptying (min^−1^), *f*: fraction of absorbed ethanol.

Single-compartment first-order model of ethanol absorption
$\frac{dG}{dt}=-k_{A}\cdot G\left(t\right),\;G\left(0\right)=dose\;of\;consumed\;ethanol$
Single-compartment first-order model of ethanol absorption adjusted for absorbed fraction
$\frac{dG}{dt}=-k_{A}\cdot (G\left(t\right)-\left(1-f\right)\cdot G\left(0\right)),\;G\left(0\right)=dose\;of\;consumed\;ethanol$
Two-compartment first-order model of ethanol absorption
$\frac{dG}{dt}=-k_{G}\cdot G(t),\;G\left(0\right)=dose\;of\;consumed\;ethanol$ $\frac{dI}{dt}=-\frac{dG}{dt}-k_{A}\cdot I\left(t\right),\;I\left(0\right)=0$
Two-compartments first-order model of ethanol absorption adjusted for absorbed fraction
$\frac{dG}{dt}=-k_{G}\cdot \left(G\left(t\right)-\left(1-f\right)\cdot G\left(0\right)\right),\;$ $G\left(0\right)=dose\;of\;consumed\;ethanol$ $\frac{dI}{dt}=-\frac{dG}{dt}-k_{A}\cdot I\left(t\right),\;I\left(0\right)=0$

### Appendix A.2. Distribution

In classical mathematical models of ethanol pharmacokinetics, its distribution in only one compartment is assumed [20,22]. The volume of this compartment is equal to the volume of distribution of ethanol (VD) and can be approximated with the TBW. However, the solubility of ethanol differs between individual tissues, as does the perfusion of the tissues with blood. Therefore, the two-compartment ethanol distribution model (Figure A1) is more appropriate because the change in ethanol concentration in the blood at an early stage after consumption is not only a consequence of elimination but also of distribution between the two compartments [22].

The change in ethanol concentration in the central and peripheral compartments due to redistribution between compartments is given by the following system of differential equations:

$$\frac{dC_{C}}{dt}=\frac{k_{CP}{\cdot (C}_{P}\left(t\right)-C_{C}\left(t\right))}{V_{C}}$$

$$\frac{dC_{P}}{dt}=\frac{k_{CP}{\cdot (C}_{C}\left(t\right)-C_{P}\left(t\right))}{V_{P}}$$

In the above system of differential equations, *k_CP_* is the distribution constant between compartments expressed in L/min. Steady state is reached when the ethanol concentrations in the central and peripheral compartments are equal.

In the recently published mathematical models of ethanol pharmacokinetics, the liver, the major site of ethanol elimination, is modelled as a separate compartment [23] (Figure A2).

### Appendix A.3. Elimination

Ethanol is largely eliminated by oxidative metabolism in the liver, so other pathways of elimination may be neglected in mathematical models of ethanol pharmacokinetics. All enzyme systems including ADH show saturation kinetics considering the limited amount of particular enzyme in the body [2]. The rate of elimination of a substance by such enzyme systems is given by the Michaelis–Menten equation:

$$elimination\;rate=\frac{v_{MAX}\cdot C\left(t\right)}{K_{M}+C\left(t\right)}$$

In the above equation, the rate of substance elimination is expressed in g/min. *C*(*t*) represents the substance concentration at time t in g/L, *v_MAX_* is the maximum elimination rate expressed in g/min, and *K_M_* Michaelis–Menten constant expresses the substance concentration at which the enzyme system metabolizes the substance at half of the maximum elimination rate. The Michaelis–Menten equation can be simplified depending on the relative substance concentration relative to *K_M_* (Table A2).

**Table A2:** Simplification of the Michaelis–Menten equation depending on the relative concentration of the substance in relation to *K_M_* [19].

Simplification of Michaelis–Menten equation at low substance concentration
$\frac{v_{MAX}\cdot C\left(t\right)}{K_{M}+C\left(t\right)}\approx \frac{v_{MAX}\cdot C\left(t\right)}{K_{M}}=K^{\prime }\cdot C\left(t\right),\;K_{M}\gg C\left(t\right)$
Simplification of Michaelis–Menten equation at high substance concentration
$\frac{v_{MAX}\cdot C\left(t\right)}{K_{M}+C\left(t\right)}\approx v_{MAX},\;K_{M}\ll C\left(t\right)$

Table A2 implies that substances whose concentrations are significantly lower than the *K_M_* of the corresponding enzyme are seemingly metabolized by first-order kinetics, while substances whose concentrations are significantly higher than the *K_M_* are seemingly metabolized by zero-order kinetics.

Results of previous studies have shown that ADH *K_M_* is between 0.08 and 0.1 g/L [2]. Therefore, for blood ethanol concentrations above 0.1 g/L (which is lower than concentrations achieved with moderate ethanol consumption) it is appropriate to assume that ethanol elimination is linear. Hence, the classic model used in pharmacokinetic and forensic studies of ethanol is the zero-order Widmark model [5]:

$$\frac{dC}{dt}=-\beta ,\;C\left(0\right)=C_{0}$$

In the differential equation above *C*(*t*) represents the ethanol concentration in the blood in g/L, *β* is the rate of change of the blood ethanol concentration in g/(L × min), and its solution is given by:

$$C\left(t\right)=C_{0}-\beta \cdot t$$
